# Clinical and genetic determinants of survival in amyotrophic lateral sclerosis patients from North India

**DOI:** 10.1093/braincomms/fcag003

**Published:** 2026-01-08

**Authors:** Shiffali Khurana, Mandaville Gourie-Devi, Yuvraj Vats, Sagar Verma, Nirmal Kumar Ganguly, Parul Chugh, Ankkita Sharma, Laxmi Khanna, Uma Dhawan, Vibha Taneja

**Affiliations:** Department of Biotechnology and Research, Sir Ganga Ram Hospital, Delhi, India, 110060; Department of Biomedical Science, Bhaskaracharya College of Applied Sciences, University of Delhi, Delhi, India, 110075; Department of Neurophysiology, Sir Ganga Ram Hospital, Delhi, India, 110060; Department of Neurology, Sir Ganga Ram Hospital, Delhi, India, 110060; Department of Biotechnology and Research, Sir Ganga Ram Hospital, Delhi, India, 110060; Department of Biotechnology and Research, Sir Ganga Ram Hospital, Delhi, India, 110060; Department of Biotechnology and Research, Sir Ganga Ram Hospital, Delhi, India, 110060; Department of Biotechnology and Research, Sir Ganga Ram Hospital, Delhi, India, 110060; Department of Neurophysiology, Sir Ganga Ram Hospital, Delhi, India, 110060; Department of Neurophysiology, Sir Ganga Ram Hospital, Delhi, India, 110060; Department of Biomedical Science, Bhaskaracharya College of Applied Sciences, University of Delhi, Delhi, India, 110075; Department of Biotechnology and Research, Sir Ganga Ram Hospital, Delhi, India, 110060

**Keywords:** amyotrophic lateral sclerosis, survival, clinical and genetic heterogeneity, whole-exome sequencing

## Abstract

Amyotrophic lateral sclerosis (ALS) is a fatal neurodegenerative disease characterized by progressive motor neuron degeneration, with significant clinical and genetic variability. While the role of genetic factors is well-established in ALS pathogenesis, their impact on survival outcomes remains poorly understood, particularly in the Indian population. We performed whole-exome sequencing in 159 ALS patients from North India (familial = 2, sporadic = 157). Clinical parameters, including age at onset, site of onset, sex, family history and survival, were recorded. Males exhibited shorter survival than females, but did not achieve statistical significance (median: 48 versus 60 years, *P* = 0.05). Bulbar-onset patients developed ALS at a significantly older age (mean: 59.7 versus 54 years, *P* = 0.007) and experienced poorer survival outcomes than spinal-onset patients (median: 48 versus 60 months, *P* = 0.03). A small subset of ALS patients (6.3%, *n* = 10) had very long survival duration of more than 10 years. We identified 102 genetic variants in 92 ALS patients, of which 45 variants were novel. According to American College of Medical Genetics and Genomics guidelines, 13.5% of total variants were pathogenic, 19.8% were likely pathogenic, and 66.7% were variants of uncertain significance. The presence of genetic variations was significantly associated with delayed onset (mean: 53.4 versus 57.1 years, *P* = 0.049) and diminished life expectancy (median: 48 versus 60 months, *P* = 0.029). Variations in more than one gene were detected in 16.7% of the patients, supporting the theory of oligogenic basis for ALS. After adjusting for age at onset, increased risk of mortality was associated with males [hazard ratio = 1.740, 95% confidence interval (CI) = 1.105–2.740] and rare genetic variations (hazard ratio = 1.533, 95% CI = 1.001–2.350). Furthermore, bulbar onset (hazard ratio = 1.75, 95% CI = 1.11–2.75) was found to be a negative prognostic factor for survival. Our study provides valuable insights into the genetic complexity and its impact on clinical outcomes in ALS patients of North Indian origin.

## Teaser

Khurana *et al*. identified deleterious genetic variants in 56.8% of sporadic amyotrophic lateral sclerosis (ALS) patients and two familial ALS cases. About 16.7% had more than one deleterious variant, suggesting genetic interactions influence disease risk and progression. The study also found that males and patients with bulbar onset are at higher risk for ALS.

## Graphical Abstract

**Graphical Abstract:**
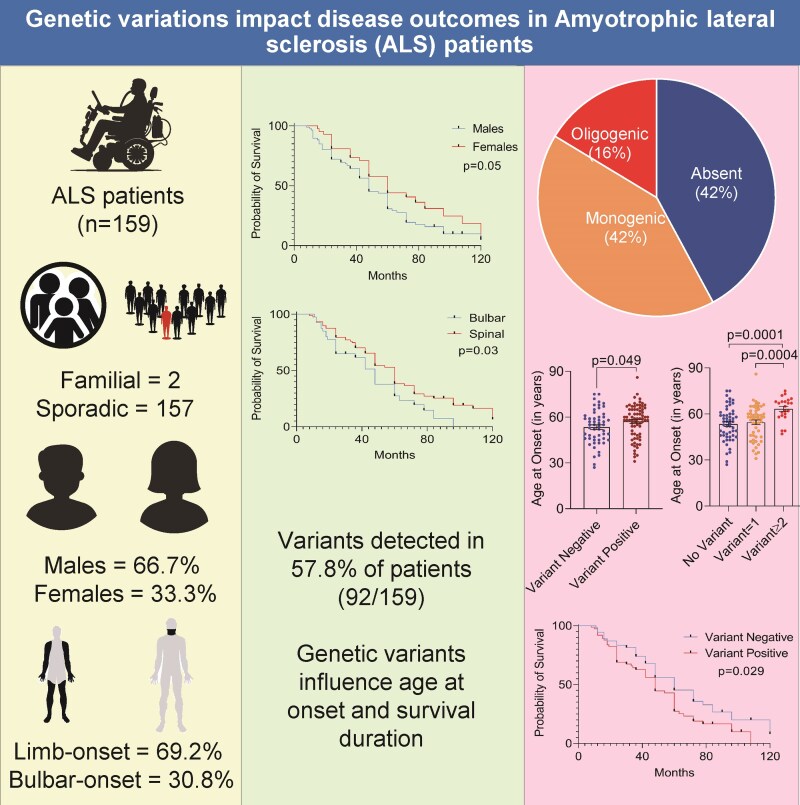

## Introduction

Amyotrophic lateral sclerosis (ALS) is a devastating and rapidly progressive neurodegenerative disorder that affects the upper and lower motor neurons in the motor cortex, brainstem and spinal cord. Clinically, ALS manifests as progressive muscular weakness, atrophy followed by paralysis and eventually death due to respiratory arrest. The median survival for ALS patients is typically 2–5 years from the onset of symptoms, though survival outcomes vary widely, ranging from a few months to over a decade.^1-3^ Several clinical features including the site of onset of symptoms, age at onset, diagnostic delay, respiratory dysfunction, cognitive and behavioural deficits and environmental factors may affect the disease progression and survival in ALS patients, underpinning the multifaceted nature of ALS.^4-7^ Moreover, this variability has been suggested to be influenced by inter-individual genetic differences.^6^

Familial ALS (fALS) accounts for ∼5–10% of ALS cases, whereas sporadic ALS (sALS) constitutes ∼90–95% of the cases.^8^ However, in India, the occurrence of fALS is extremely rare, accounting for <1% of cases.^2,9^ Mutations in superoxide dismutase 1 (SOD1), TAR DNA-binding protein (TARDBP), fused in sarcoma (FUS), and hexanucleotide repeat expansion in chromosome 9 open reading frame 72 (C9orf72) represent the most frequent genetic causes of ALS.^10,11^ Variations in these genes are further associated with decreased survival.^12-15^ However, certain SOD1 mutations such as L144S and D91A have been linked to relatively slower disease progression and increased survival.^16-19^ In addition to variations in disease-causative genes, variations in disease-modifying genes have also been suggested to impact the phenotypic expression of ALS, adding to the complexity of genetic influence in ALS.^11^ Furthermore, the presence of multiple rare variants within a single individual has been reported to have synergistic effects on disease presentation, suggesting the oligogenic nature of ALS.^20,21^

Multiple studies have explored the mechanisms driving disease onset and progression, but the relationship between ALS genotypes and phenotypes remains elusive. Here, we screened genetic variations in ALS-causative, susceptibility and disease-modifier genes and assessed their impact on survival outcomes in ALS patients. Furthermore, there is a considerable clinical and genetic overlap between ALS and other neurodegenerative disorders (NDD) or neuromuscular disorders (NMD).^22-28^ To address this genetic pleiotropy, genes associated with NDD and NMD were also examined. By delving deeper into the mutation spectrum, this study aims to better understand their correlation with symptom manifestation and disease progression, ultimately paving the way for more personalized prognoses and treatment strategies for ALS patients.

## Materials and methods

### Patient enrolment and clinical assessment

A total of 159 ALS patients were recruited at Sir Ganga Ram Hospital, Delhi, between September 2013 and December 2022. Written informed consent was taken from all the patients. The study was approved by the Institutional Ethics Committee of Sir Ganga Ram Hospital (EC/05/20/1714) and was conducted in accordance with the Declaration of Helsinki. The study was conducted according to STrengthening the REporting of Genetic Association Studies (STREGA) guidelines.^29^

Patients were diagnosed based on comprehensive evaluation, including (i) detailed history and neurological examination; (ii) motor nerve conduction of median and ulnar nerves (upper limbs) and peroneal and posterior tibial nerves (lower limbs); (iii) sensory nerve conduction of median, ulnar and sural nerves; (iv) electromyography of distal and proximal muscles of all four limbs, bulbar and paraspinal muscles; and (v) magnetic resonance imaging of the brain and spinal cord to exclude other neurological disorders.

Based on the revised El Escorial criteria, patients diagnosed as ‘Clinically Definite ALS’ and ‘Clinically Probable ALS’ were included. ‘Clinically probable ALS-Laboratory supported’, ‘Clinically possible ALS’ and ‘Clinically Suspected ALS’ patients were excluded.^30^

Clinical and demographic features including a detailed illness history with emphasis on the site of onset, symptoms suggestive of lower motor neuron involvement (atrophy, weakness manifesting as difficulty in daily activities), upper motor neuron involvement (stiffness of limbs, difficulty in walking), bulbar involvement (speech and swallowing difficulties), pseudobulbar (emotional lability), age at onset, sex distribution and family history were recorded for all the patients. The period of survival was determined from the onset of symptoms to (i) last follow-up (15th November 2024) or (ii) tracheostomy or (iii) death. As on the last date of contact, patients who were alive (40), deceased (98) or lost to follow-up (21) were included in the study.

### Whole-exome sequencing

Genomic DNA was extracted from peripheral blood and whole-exome sequencing (WES) was carried out in ALS patients using NEBNext® Ultra™ II DNA Library Prep Kit (Cat No. E7645). DNA libraries were indexed, pooled and sequenced on the Illumina Novaseq 6000 Platform, with an average read depth of 48× and ranged from 39× to 78×. Data obtained was de-multiplexed, followed by adaptor trimming and quality filtering using the fastp tool. Low-quality bases (Phred score <15), poly-G and poly-X bases were removed from the 3′ end, and reads ≥50 bp were retained post-trimming. High-quality reads were mapped to the human genome reference (hg19), using Agilent SureCall tool version 4.2.2. Read targeting and variant calling were performed using Agilent Standard user guidelines, generating Variant Call Format (VCF) files. The VCF files were annotated using the ANNOVAR software tool (http://wannovar.wglab.org/).

### Selection of genes and variant identification

Based on the Amyotrophic Lateral Sclerosis Online Database (http://alsod.iop.kcl.ac.uk/), genes were classified into causative (*n* = 17), susceptibility (*n* = 21) and disease-modifier (*n* = 126). Rare pathogenic variations in an additional set of 217 genes associated with NDD/NMD were also investigated (Supplementary Table S1).^31^

**Figure 1:**
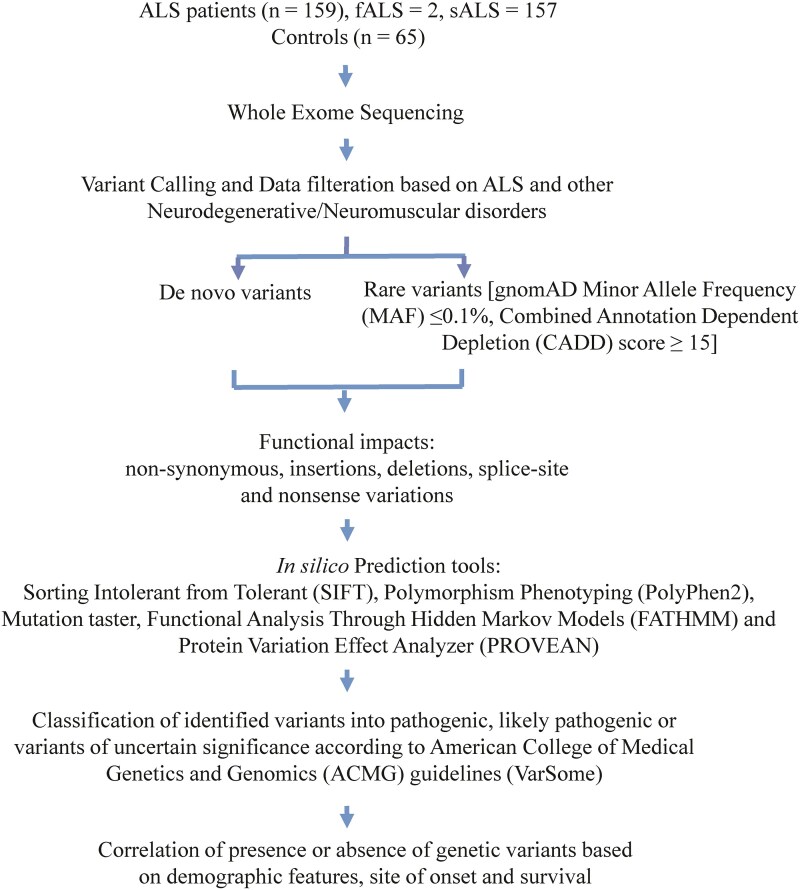
**Workflow for identification of genetic variants**.

The prioritization of genetic variations was done by the following criteria: (i) non-synonymous, insertions, deletions, splice-site and nonsense variations with genotype quality score ≥25 and (ii) variants with minor allele frequency (MAF) < 0.01% in Genome Aggregation Database (gnomAD) and Combined Annotation Dependent Depletion (CADD) score >15. Functional consequences and deleteriousness of prioritized variants were assessed by prediction algorithms including, SIFT (https://sift.bii.a-star.edu.sg/), PolyPhen2 (http://genetics.bwh.harvard.edu/pph2/), Mutation taster (https://www.mutationtaster.org/ChrPos.html), FATHMM (https://fathmm.biocompute.org.uk/) and Provean (https://www.jcvi.org/research/provean). Identified variants were classified according to American College of Medical Genetics and Genomics (ACMG) guidelines into pathogenic, likely pathogenic, and variants of uncertain significance (VUS) using Varsome database (https://varsome.com/). The workflow for identification of genetic variants is outlined in Fig. 1.

### Statistical analysis

Descriptive statistics for continuous variables (age at onset and survival duration) are presented as mean ± SEM or median (IQR); categorical variables (sex and site of onset) are presented as frequencies and percentages. Chi-square test was used to explore differences between groups for categorical data. As the variables were not normally distributed (tested with Kolmogorov Smirnov), Mann–Whitney U or Kruskal–Wallis tests were used to find if the differences between two or more groups are significant. For multiple testing corrections, Benjamini–Hochberg correction was performed. Survival analysis was done using the log-rank Mantel-Cox test, displayed as Kaplan–Meier curves. Data were analysed using GraphPad Prism Software version 10.4.0.

The relationship of each independent variable, including sex, age at onset, site of onset, and genetic variations with survival was initially assessed using univariate Cox regression analysis to estimate hazard ratios (HRs) and 95% confidence intervals (CIs). A forward stepwise approach was applied for multivariate Cox regression, using a retention criterion of *P* < 0.1 for the independent variables. A *P*-value < 0.05 was considered statistically significant. Data were analysed using the SPSS statistical software, version 22.0 (IBM Corporation, Chicago, IL, USA).

## Results

### Demographic and clinical characteristics of ALS patients

Demographic and clinical features of 159 ALS patients (familial = 2, sporadic = 157), stratified based on sex, are listed in Table 1. The mean age at disease onset was 55.31 ± 10.83 years, with a male-to-female ratio of 2:1. To investigate any correlation between age at disease onset, sex, site of onset and survival, patients were categorized into four groups based on survival duration^32,33^: short (≤24 months), average (25–60 months), long (61–120 months) and very long (>120 months) (Table 2). No significant association was found between age at onset and survival duration (*P*  *= 0.634*), as confirmed by univariate analysis (HR = 1.009, 95% CI = 0.990–1.030, *P* = *0.355*, Supplementary Table S2A). Kaplan–Meier survival analysis indicated shorter survival among patients with age at onset >65 years compared to those between 46–65 years and 0–45 years of age; however, the difference did not reach statistical significance (*P = 0.478*, Supplementary Fig. 1A).

**Table 1:** Demographic features of ALS patients based on sex

	Total	Males	Females	*P*-value
Number of patients, *n* (%)	159	106 (66.7%)	53 (33.3%)	
Age at onset, mean ± SD	55.31 ± 10.83	55.02 ± 11.14	55.79 ± 10.19	0.379
Site of onset				
Bulbar, *n* (%)	49 (30.8%)	27 (25.5%)	22 (41.5%)	**0.046**
Spinal, *n* (%)	110 (69.2%)	79 (74.5%)	31 (58.4%)	
Survival duration (in months), median (IQR)	48.0 (24.0–72.0)	48 (24.0–72.0)	60.0 (36.0–78.0)	
Survival status				
Deceased, *n* (%)	98 (61.6%)	68 (64.1%)	30 (56.6%)	0.652
Alive, *n* (%)	40 (25.2%)	25 (23.6%)	15 (28.3%)	
Lost to follow-up, *n* (%)	21 (13.2%)	13 (12.3%)	8 (15.1%)	
Variation				
Present	92 (60.4%)	61 (57.5%)	31 (58.4%)	0.99
Absent	67 (39.6%)	45 (42.5%)	22 (41.5%)	

Bold *P*-values are statistically significant. *P*-values are based on the chi-square test.

**Table 2:** Demographic features of ALS patients based on survival duration

	Short (≤24 months)	Average (25–60 months)	Long (61–120 months)	Very long (>120 months)	*P*-value
Number of patients, *n* (%)	37 (23.3%)	59 (37.1%)	32 (20.1%)	10 (6.3%)	
Age at onset, mean ± SD	57.68 ± 11.03	54.93 ± 11.90	54.34 ± 9.64	55.50 ± 6.49	0.634
Sex					
Male, *n* (%)	28 (75.7%)	39 (66.1%)	19 (59.4%)	7 (70%)	0.538
Female, *n* (%)	9 (24.3%)	20 (33.9%)	13 (40.6%)	3 (30%)	
Site of onset					
Bulbar, *n* (%)	17 (46%)	16 (27.1%)	7 (21.9%)	3 (30%)	0.137
Spinal, *n* (%)	20 (54%)	43 (72.9%)	25 (78.1%)	7 (70%)	
Survival duration (in months), median (IQR)	12.0 (10.5–12.0)	42 (24.0–48.0)	84 (72.0–99.0)	174 (144.0–252.0)	
Survival status					
Deceased, *n* (%)	32 (86.5%)^a,d^	44 (74.6%)^b,c^	20 (62.5%)^a,b,e^	2 (20%)^c,d,e^	**0.0004**
Alive, *n* (%)	5 (13.5%)	15 (25.4%)	12 (37.5%)	8 (80%)	
Variation					
Present	28 (75.7%)^a^	33 (55.9%)	13 (40.6%)^a^	6 (60%)	**0.031**
Absent	9 (24.3%)	26 (44.1%)	19 (59.4%)	4 (40%)	

Bold *P*-values are statistically significant. *P*-values are based on chi-square test.

a, b, c, d and e denote statistical significance achieved between corresponding subgroups.

In our dataset, 6.3% of ALS patients (*n* = 10) exhibited exceptionally long survival durations (>10 years). The inclusion of these cases could significantly impact the overall survival analysis and skew the data. Hence, for a more focused and meaningful assessment of the survival patterns in ALS, we have excluded these patients from the survival analysis. Genetic variations in these patients are detailed in Supplementary Table S3.

There was no significant difference in age at onset between males and females (*P* = *0.379*, Table 1). Although males exhibited shorter survival compared to females, this difference did not reach statistical significance (median: 48 versus 60 years, *P* = *0.05*, Supplementary Fig. 1B) and was not corroborated in univariate analysis (HR = 1.498, 95% CI = 0.967–2.321, *P* = *0.071*, Supplementary Table S2A).

Furthermore, we observed that patients with bulbar onset developed the disease at a significantly older age (*P* = 0.007, Supplementary Fig. 1C) and had a shorter median survival duration (48 months) than spinal-onset patients (60 months, *P*  *= 0.03*, Supplementary Fig. 1D). This observation was further supported by univariate analysis, which revealed that bulbar onset was significantly associated with an increased hazard of mortality (HR = 1.565, 95% CI = 1.009–2.421, *P*  *= 0.045*, Supplementary Table S2A).

Multivariate cox-proportional hazard analysis identified males (HR = 1.740, 95% CI = 1.105–2.740, *P = 0.017*) and the presence of genetic variations (hazard ratio = 1.533, 95% CI = 1.001–2.350, *P = 0.05*) as significant predictors of higher risk of mortality. Additionally, bulbar onset (hazard ratio = 1.75, 95% CI = 1.11–2.75, *P = 0.016*) was identified as a negative prognostic factor for survival (Supplementary Table S2B).

### Identification of genetic variants

**Figure 2:**
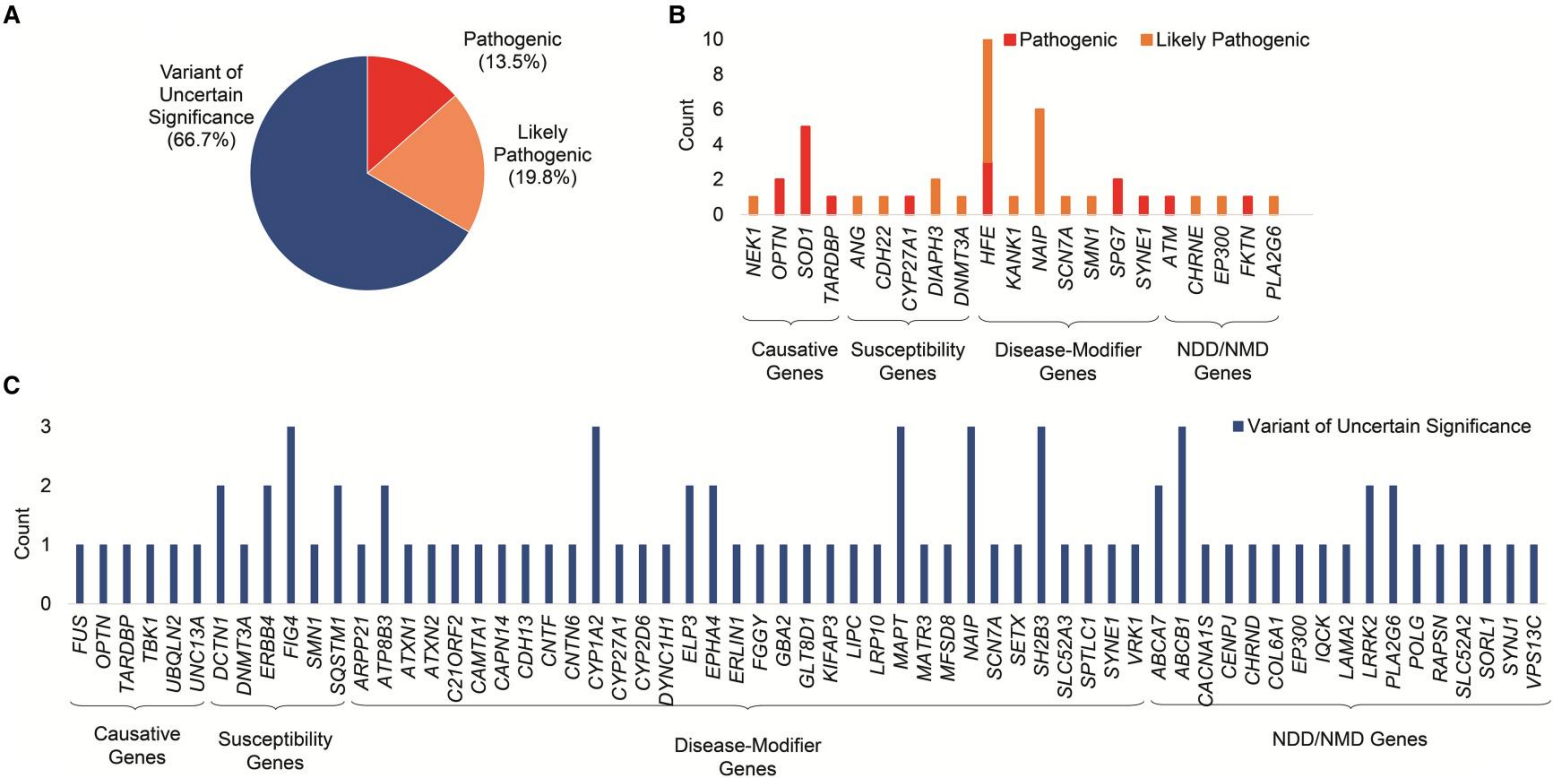
**Summary of identified variants on the basis of ACMG guidelines (Varsome) and correlation with clinical parameters.** (**A**) Pie-chart illustrates the classification of genetic variants: pathogenic, likely pathogenic and variant of uncertain significance. (**B**) and (**C**) Bar charts display the counts of pathogenic (in red), likely pathogenic (in orange) and variants of uncertain significance (in blue) identified in primary ALS genes, disease-modifier genes and NDD/NMD-associated genes within our subset. The genes are arranged in alphabetical order for each gene subcategory.

WES identified a total of 102 genetic variations in 57.9% (*n* = 92) of ALS patients, whereas 42.1% (*n* = 67) of the patients showed no genetic variations. Most (*n* = 88) of the variants were non-synonymous, 12 were nonsense, and 2 were deletions. No splice-site variants were identified. Remarkably, 45 of these variants appear to be novel [marked with asterisk (*) in Supplementary Tables S3–S5] and have not been documented previously in the literature. Among the identified variations, 12 variants were detected in 9 causative ALS genes, 14 variants in 8 susceptibility genes, 47 variants in 38 disease-modifier genes and 29 variants in 21 genes associated with NDD/NMD (Supplementary Tables S3–S5). According to ACMG guidelines, 13.5% of all the variations were classified as pathogenic, 19.8% as likely pathogenic, and 66.7% were VUS (Fig. 2A). Genes with pathogenic, likely pathogenic variants, and VUS are depicted in Fig. 2B and C. Pathogenic variants were present in 10% (*n* = 16) patients, likely pathogenic in 15% (*n* = 24) patients and VUS in 42% (*n* = 67) patients. Among these, 26 patients had more than one variation (Supplementary Table S5).

Furthermore, among the causative ALS genes, three pathogenic variants were detected in *SOD1*. Pathogenic as well as VUS were detected in *TARDBP and OPTN.* Additionally, a likely pathogenic variant in *NEK1* and a VUS in *TBK1* gene were identified. Among the susceptibility genes, pathogenic variants were detected in *DNMT3A*, and likely pathogenic variants were identified in *CDH22* and *ANG. SMN1* and *DNMT3A* harboured both likely pathogenic variants and VUS. Furthermore, VUS were detected in *SQSTM1*, *FIG4* and *ERBB4*.

Analysis of disease-modifier genes revealed pathogenic as well as likely pathogenic variants in *HFE*, pathogenic variants in *SPG7*, pathogenic variants and VUS in *CYP27A1* and *SYNE1*. Additionally, likely pathogenic variants and VUS were identified in *NAIP* and *SCN7A*. The VUS were found in 30 genes including *EPHA4*, *DCTN1*, *MAPT*, *SETX*, MATR3, ERLIN1 and CAMTA1.

In genes associated with other NDD or NMD, *ATM* and *FKTN* variants were pathogenic, *CHRNE* variant was likely pathogenic, *PLA2G6* and *EP300* variants were likely pathogenic as well as VUS. All other identified variants in NDD or NMD genes were VUS. The details of all identified variants are provided in Supplementary Tables S3–S5.

### Correlation of clinical parameters with the presence or absence of genetic variations

**Figure 3:**
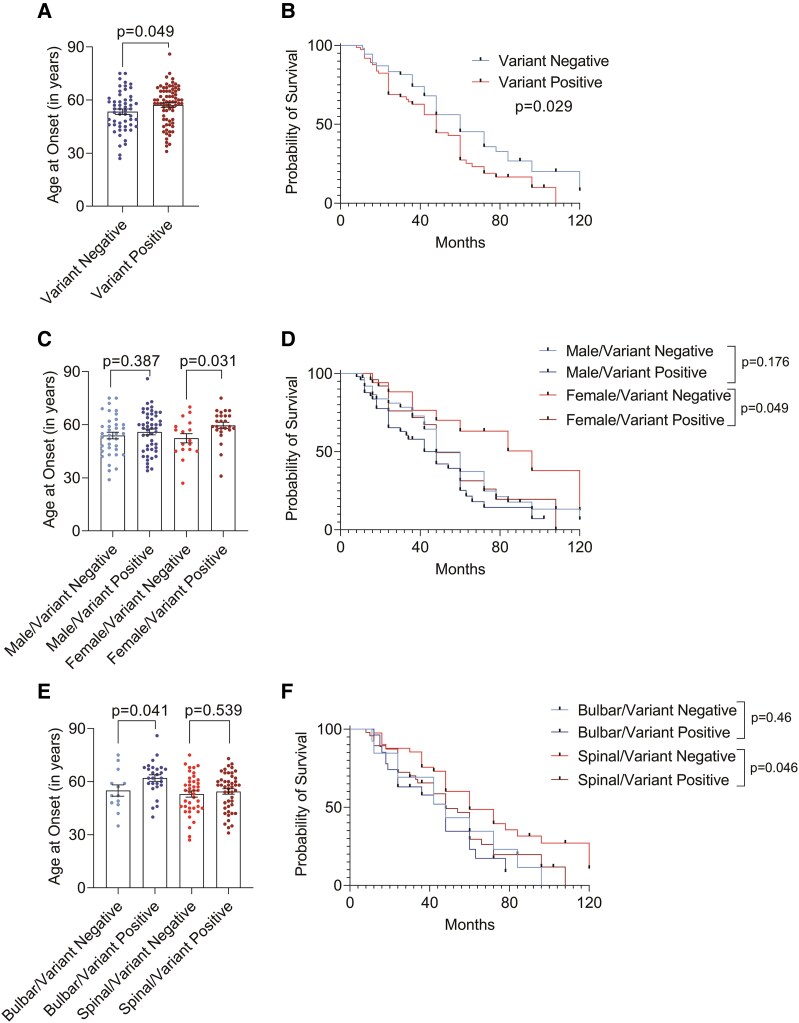
**Correlation of clinical parameters with presence or absence of genetic variations.** (**A**) Age at onset in patients negative and positive for genetic variations. (**B**) Kaplan–Meier survival analysis of patients negative and positive for genetic variations. (**C**) Age at onset in males and females with genetic variations. (**D**) Kaplan–Meier survival analysis of males and females with genetic variations. (**E**) Age at onset in bulbar- and spinal-onset patients with genetic variations. (**F**) Kaplan–Meier survival analysis of bulbar- and spinal-onset patients with genetic variations. Description of tests used for statistical analysis. (**A**, **B**) Variant negative *n* = 54; variant positive *n* = 74, *P = 0.049* in Mann–Whitney U-test, *P* = 0.029 in log-rank test. (**C**, **D**) Male/variant negative *n* = 37, male/variant positive *n* = 49; female/variant negative *n* = 17; female/variant positive *n* = 25, *P = 0.387* and *P = 0103* in ANOVA; male/variant negative versus male/variant positive *P = 0.387*; female/variant negative versus female/variant positive *P* = 0.031, *P* = *0.176* and *P = 0.049* in log-rank test for male/variant negative versus male/variant positive and for female/variant negative versus female/variant positive, respectively. (**E**, **F**) Bulbar/variant negative *n* = 13; bulbar/variant positive *n* = 27; spinal/variant negative *n* = 41; spinal/variant positive *n* = 47, *P = 0.036* in ANOVA; bulbar/variant negative versus bulbar/variant positive *P = 0.041*; spinal/variant negative versus spinal/variant positive *P* = 0.539, *P* = *0.46 and P = 0.046* in log-rank test for bulbar/variant negative versus bulbar/variant positive and for spinal/variant negative versus spinal/variant positive, respectively. A *P*-value of ≤0.05 was considered as statistically significant. One-way ANOVA analysis followed by Benjamini–Hochberg corrections was performed for multiple group comparisons. Kaplan–Meier curves and log-rank tests were applied to determine the effect of demographic or variation status on survival. Individual data points represent the number of patients (*n*) for each group.

Patients (*n* = 74) harbouring genetic variations showed a significant delay in the disease onset (*P* = *0.049,* Fig. 3A) compared to patients (*n* = 54) with no genetic variations. Kaplan–Meier survival analysis revealed a shorter median disease duration in patients with genetic variations (48 months) as compared to those without genetic variations (60 months, *P* = *0.029*, Fig. 3B).

When data were stratified based on sex, females with genetic variations had a significantly later disease onset (*P* = *0.031*, Fig. 3C) and a shorter median survival duration (60 months) compared to females without genetic variations (96 months, *P* = *0.049*, Fig. 3D). In contrast, no significant differences in disease onset (*P* = 0.387, Fig. 3C) or survival duration (*P = 0.176*, Fig. 3D) were observed among males, who showed median survival of 48 months irrespective of the presence of genetic variations.

In patients with bulbar onset and positive for genetic variations, disease onset occurred at a later age (*P*  *= 0.041*, Fig. 3E) and shorter median survival duration (30 months) compared to those without genetic variations (48 months, *P*  *= 0.46*, Fig. 3F). However, in spinal-onset patients, the presence of genetic variations was significantly associated with a shorter median survival (48 months versus 60 months; *P = 0.046*; Fig. 3F). Overall, the presence of genetic variations demonstrated a tendency to negatively impact survival outcomes.

**Figure 4:**
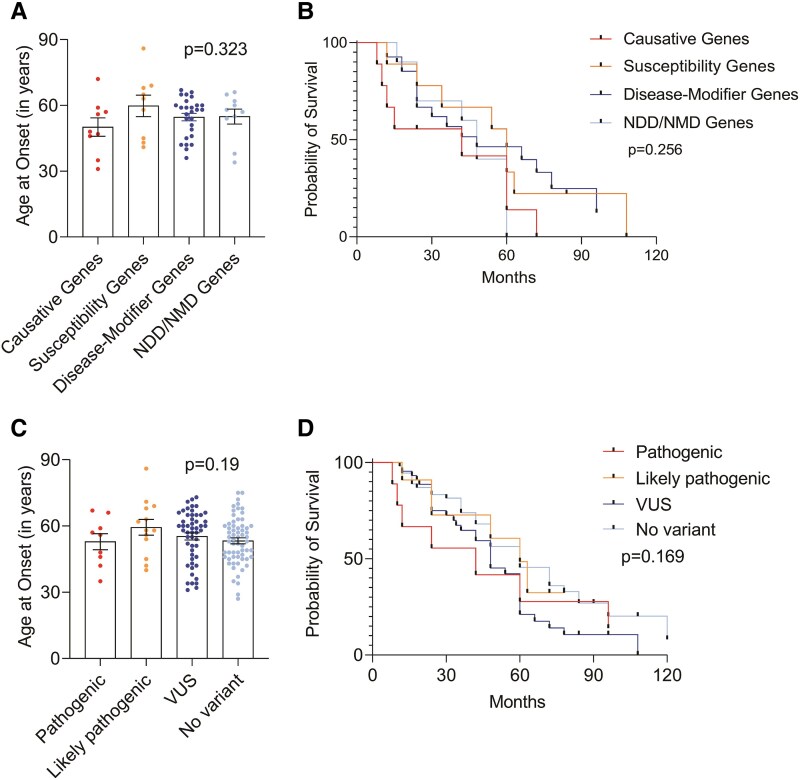
**Clinical correlation of patients stratified by variants identified in different genes with (A) age at onset, (B) Kaplan–Meier survival analysis, (C) correlation of pathogenicity of variants with age at onset and (D) Kaplan–Meier survival analysis.** Description of tests used for statistical analysis. (**A**, **B**) Causative ALS genes *n* = 10, susceptibility genes *n* = 9, disease-modifier genes *n* = 26, NDD/NMD genes *n* = 10, *P* = 0.8473 in ANOVA and *P* = 0.3854 in the log-rank test. (**C**, **D**) Pathogenic *n* = 27, likely pathogenic *n* = 85, VUS *n* = 26, no variant *n* = 54, *P* = 0.316 in ANOVA and *P* = 0.693 in log-rank test. NDD, neurodegenerative disorder, NMD, neuromuscular disorder, VUS, variants of uncertain significance. A *P*-value of ≤0.05 was considered as statistically significant. Individual data points represent the number of patients (*n*) for each group.

Furthermore, stratification of patients based on gene subcategories, namely, causative, susceptibility, disease-modifier or NDD/NMD genes, revealed no significant differences in mean age at onset (*P*  *= 0.323*, Fig. 4A). The corresponding median survival durations were 42, 60, 48 and 48 months, respectively, which also did not show statistical significance (*P* = *0.256*; Fig. 4B). Similarly, when analysed by variant pathogenicity, no significant differences were observed in either mean age at onset (*P* = 0.19; Fig. 4C) or survival duration. Median survival was 42 months for patients with pathogenic variants, 60 months for likely pathogenic variants, 48 months for variants of uncertain significance (VUS) and 60 months for those without any variant, with no statistically significant difference (*P = 0.169*; Fig. 4D).

### Multiple genetic variants influence the age at onset and survival duration

**Figure 5:**
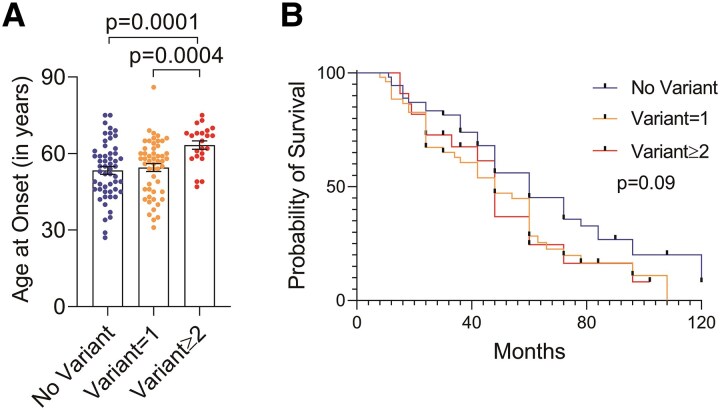
**Clinical correlation of multiple genetic variations (A) correlation of age at onset (B) Kaplan–Meier survival in patients stratified by the absence of genetic variants and presence of either a single variant or multiple (≥2) genetic variants.** Description of tests used for statistical analysis. (**A**, **B**) No variant *n* = 54; variant = 1 *n* = 74; variant ≥ 2 *n* = 22, *P* = *0.0003* in ANOVA; no variant versus variant ≥ 2, *P* = *0.0001*; variant = 1 versus variant ≥ 2, *P* = *0.0004*. One-way ANOVA analysis followed by Benjamini–Hochberg corrections was performed for multiple group comparisons. *P* = *0.09* in log-rank test. Individual data points represent the number of patients (*n*) for each group.

Interestingly, 16.7% of patients (*n* = 25) harboured more than one genetic variation. The mean age at onset was significantly delayed (63.4 years) compared to those with single variation (*n* = 52, 54.5 years, *P*  *= 0.0004*) or no variation (*n* = 54, 53.4 years, *P*  *= 0.0001*) (Fig. 5A). It is noteworthy that 60% of the patients with multiple variants had onset in the seventh or eighth decade (Supplementary Table S5). On the contrary, ∼70% of patients with a single variant (Supplementary Table S4) or no variant (data not shown) developed symptoms before the seventh decade. Other demographic and clinical features of the patients categorized by the absence and presence of either a single or multiple (≥2) genetic variations are detailed in Supplementary Table S6.

Among the patients with multiple genetic variations, 15 patients carried a combination of pathogenic or likely pathogenic variants along with VUS, while 10 patients had more than one VUS. Based on gene subcategorization, eight patients with variations in causative ALS genes and two patients with susceptibility genes (*n* = 2) also carried variants in disease modifiers and NDD/NMD genes. Eight patients exhibited two distinct variants in disease-modifier genes, and five patients carried variations in both disease-modifier and NDD/NMD genes. Two patients had multiple variations specifically in NDD/NMD genes (Supplementary Table S5).

When patients were compared for survival duration, the median survival was higher (60 months) for patients with no genetic variation than patients with single (42 months) or multiple (39 months) variations. However, this difference did not reach statistical significance (*P*  *= 0.09*, Fig. 5B).

### Genotype–phenotype correlation in patients with ALS

The genotype–phenotype correlation may have an implication in predicting the disease course and understanding the disease mechanisms. A marked clinical heterogeneity in age at onset, site of onset and disease duration, even among individuals carrying the same variant, was observed (Table 3 and Supplementary Tables S3–S5).

**Table 3:** Genetic variants and associated phenotype in patients with ALS

Gene	Patient ID	Variant	ACMG classification	Another gene (variant; classification)	Demographic features		Clinical parameters	
					Age at onset	Sex	Site of onset	Survival duration (in months)
SOD1	P79	p.H43R	P	—	48	M	Both lower limb	60
	P17	p.A4T	P	—	35	M	Right upper limb	8
	P97	p.A4T	P	—	57	M	Left lower limb	12
	P140	p.L84F	P	PLA2G6 (p.V310M; VUS)	50	F	Left lower limb	-
	P129	p.L84F	P	NEK1 (p.R738X; LP), KANK1 (p.G1234X; LP), ATP8B3 (p.P188R; VUS),CENPJ (p.G1286D; VUS)	59	M	Bulbar	15
TARDBP	P67	p.I383V	P	—	46	M	Right upper limb	10
	P136	p.M405L	VUS	EP300 (p.D32G; VUS)	47	M	Right upper limb	72
FUS	P206	p.R269W	VUS	CNTN6 (p.A871V; VUS), ERLIN1 (p.A34V; VUS)	72	M	Bulbar	24^a^
TBK1	P176	p.G272D	VUS	—	31	F	Right lower limb	60
OPTN	P111	p.E399X	P	—	56	M	Left lower limb	42
	P75	p.E399X	P	CYP2D6 (p.A305V; VUS)	75	F	Bulbar	24
	P117	p.K489E	VUS	ATM (p.S2812Vfs*3; P)	49	F	Right upper limb	48
FIG4	P158	p.I272M	VUS	—	65	F	Bulbar	60
	P164	p.A358V	VUS	ELP3 (p.D183G; VUS)	68	F	Bulbar	7
	P32	p.S168R	VUS	UNC13A (p.A662E; VUS), GLT8D1 (p.L160V; VUS)	69	M	Bulbar	48
SQSTM1	P205	p.R393W	VUS	—	58	F	Right upper limb	108
	P80	p.R393W	VUS	VPS13C (p.G3344R; VUS)	67	F	Bulbar	48
HFE	P204	p.C282Y	P	—	59	F	Bulbar	24
	P217	p.H63D	LP	—	40	M	Bulbar	30
	P218	p.H63D	LP	—	54	M	Both limb	60
	P99	p.H63D	LP	—	62	F	Bulbar	24
	192	p.H63D	LP	—	45	M	Both limb	264^a^
	200	p.H63D	LP	—	49	M	Bulbar	240^a^
	P203	p.C282Y	P	SYNE1 (p.R5617X; P), ABCB1 (p.V907F; VUS)	49	M	Left upper limb	60^a^
	P222	p.C282Y	P	CNTF (p.R72X; VUS)	58	F	Left upper limb	36^a^
	P88	p.H63D	LP	UBQLN2 (p.Q558L; VUS)	41	F	Left lower limb	—
	P196	p.C282Y	P	CYP1A2 (p.E346K; VUS)	67	M	Bulbar	36^a^

P = pathogenic; LP = likely pathogenic; VUS = variant of uncertain significance; M = male, F = female.

^a^Patients alive at the time of analysis.

Among *SOD1* carriers, the p.A4T variant was observed in two siblings with aggressive phenotype and very short survival durations. The patient with p.H43R *SOD1* variant had longer survival duration of 60 months. Two sALS patients with p.L84F *SOD1* variant carried additional variants and exhibited variable clinical features.

Two patients with variations in the *TARDBP* gene had onset in the fifth decade. Notably, one of these variations (p.I383V) is novel, classified as VUS with a CADD score >15, suggestive of its potential pathogenicity. This patient survived for 10 months after onset. The other patient with known pathogenic p.M405L variant and a VUS in *EP300* showed a longer survival duration of 72 months.

In the *OPTN* gene, well-characterized pathogenic variations, p.E399X and p.K489E, were identified in three patients with short survival durations ranging from 24 to 48 months. Additional pathogenic variant and VUS were also present in *ATM* and *CYP2D6* genes in two of these patients.

The presence of additional VUS in patients carrying variations in susceptibility genes was associated with shorter survival durations. A patient with deleterious *SQSTM1* variant (p.R393W) had prolonged survival of 108 months, whereas another patient with an additional variant in VPS13C survived only for 48 months. Similarly, a patient with a single *FIG4* variant showed a survival of 60 months, while a patient with an additional variant in *ELP3* had a markedly shorter survival of just 7 months.

Among the disease-modifier genes, two missense variations (p.H63D, *n* = 7 and p.C282Y, *n* = 3) were identified in the *HFE* gene. Six patients with p.H63D were alive at the time of analysis, and one was lost to follow-up. Two of these patients had prolonged survival of >10 years. Two patients with p.C282Y were alive while one had a shorter survival of 24 months. Four patients with these two *HFE* variants also carried deleterious variations in other disease-modifier genes (Table 3, Supplementary Table S5).

Furthermore, patients carrying variations solely in NDD genes including *LRRK2*, *ABCA7*, *ABCB1*, *SORL1* and *IQCK* had survival ≤5 years. Similar survival pattern was observed for patients with variations only in NMD genes, *CACNA1S*, *RAPSN*, *CHRND* and *LAMA2* (Supplementary Table S4).

## Discussion

This study presents a comprehensive exploration of clinical features and genetic variations and their impact on survival outcomes in ALS patients from North India. Globally, the mean age of ALS onset ranges from 51 to 66 years,^34-36^ whereas younger age at onset (46.2 ± 14.1 years) has been reported from South India.^2^ In our patient subset from North India, the mean age at disease onset was 55.31 ± 10.83 years. We observed sex-based differences in ALS presentation and prognosis, with males exhibiting poorer survival, consistent with prior reports from Italy, Brazil and China.^4,37-39^ However, contrasting studies from Spain, New Jersey and Australia have reported worse survival among females.^32,40,41^ Bulbar onset is a well-established negative prognostic factor in ALS, associated with shorter survival durations compared to spinal onset.^4,5,42-44^ Consistent with previous studies, our findings also identified bulbar onset as an independent predictor of reduced survival.

We identified both known and novel genetic variations in ALS-causative, susceptibility, disease-modifier and NDD/NMD genes in 58% of the patients. Among causative genes, *SOD1* (3.1%) and *OPTN* (1.9%) variants were frequently observed, while *FIG4* (1.9%) was the primary variant among susceptibility genes. Variants in *HFE* (6.3%) and *NAIP* (5.7%) were the most frequent among disease-modifier genes.

There are more than 150 variations in *SOD1* associated with ALS. We have previously reported p.L84F-*SOD1* mutation in a family with a history of ALS. Despite the same variation, significant variability in disease onset, progression and survival was observed among the affected family members.^9^ In the current study, two sALS patients with p.L84F variation also showed distinct clinical features and survival outcomes. The p.A4T variation identified in two fALS patients is relatively rare and associated with rapidly progressive form of fALS.^45,46^ The patient with p.H43R variant had extended survival of 60 months which contrasts with previous reports where p.H43R is associated with shorter disease duration compared to other *SOD1* mutations.^47-49^

In TARDBP gene, ∼50 missense variations have been identified which account for about 3% of fALS and <1% of sALS cases. Majority of these variations are located within the C-terminal glycine-rich domain of TDP-43, which leads to cytoplasmic mislocalization and increased tendency to form cytoplasmic inclusions.^50^ Similar to previous studies,^14,51^ we identified two variants in the C-terminal region of TARDBP, both associated with spinal-onset and marked variability in disease duration.

Optineurin (*OPTN*) binds to ubiquitinated cargo and facilitates its recruitment to the autophagosome via interaction with LC3 (microtubule-associated protein 1A/1B-light chain 3). This process is tightly regulated by TBK1, a serine/threonine kinase, which phosphorylates the ubiquitin-binding (UBA) domain of *OPTN* and *SQSTM1.* Disruption of this regulatory axis via mutations in either TBK1/OPTN or TBK1/SQSTM1 can impair autophagy. We have identified p.G272D variation in the kinase domain of Tbk1, and the ALS-associated variations in this domain have previously been shown to impair its catalytic activity.^52,53^

In the *OPTN* gene, we identified two variations, p.E399X and p.K489E, that have been previously reported in the Indian cohort.^54,55^ Moore *et al*. (2016)^56^ demonstrated that p.Q398X led to deletion of the UBA domain, preventing the recruitment of mutant protein to ubiquitinated mitochondria and attenuating mitophagy. Previously, Kamada *et al*. (2014)^57^ reported this variation in two ALS patients: one patient with bulbar onset survived for 9 years while another with upper-limb onset survived for 4 years. Furthermore, expression of this mutant in NSC-34 cells inhibited the association of Optn with myosin VI and lead to accumulation of autophagosomes, fragmentation of neuronal Golgi apparatus and activation of ER stress.^58^ The p.K489E variant has been shown to cause increased cell death and deregulate autophagy in an *in vitro* cell model of ALS.^59^

Similarly, mutations in the C-terminal UBA domain of *SQSTM1*^60^ impair its function by reducing phosphorylation and compromising ubiquitinated cargo binding and clearance. In our study, the p.R393W variant identified within this domain may disrupt SQSTM1 function and contribute to ALS pathogenesis.

*FIG4* plays a critical role in retrograde trafficking of endosomal vesicles, and variations in *FIG4* have been associated with longer disease duration in ALS.^61-63^ In our study, one patient with only FIG4 variant had survival duration of 60 months. However, patients with additional variations had survival duration of <5 years. Importantly, a patient with variations in *FIG4* and *ELP3* genes had particularly short survival of 7 months. Overexpression of *ELP3* has been suggested to be neuroprotective and reduce toxicity associated with mutant *SOD1* and *C9orf72* repeat expansion.^64^ Mutation in ELP3 has been shown to cause decreased survival in ALS and FTD patients with *C9orf72* repeat expansion.^65^ The co-occurrence of *FIG4* with *SETX* (p.L158V) or *C9orf72* repeat expansions has also been linked with poor prognosis.^66^ The co-presence of *FIG4* and *ELP3* variants in our patient might have resulted in shorter survival duration..

Multiple studies have reported significant correlation between p.H63D variant of HFE and ALS risk.^67-70^ The p.H63D variant has been shown to cause reduced SOD1 levels and increase disease duration in sALS patients.^71^ More recently, Canosa *et al*. (2023)^72^ demonstrated that patients harbouring *SOD1* mutations along with p.H63D *HFE* variant exhibited significantly longer survival durations. Notably, in our cohort, we observed two patients with exceptionally prolonged survival, which further supports the potential protective influence of the p.H63D variant in the context of ALS. Since most of the patients with HFE variants were alive at the time of analysis, follow-up of disease progression in these patients may aid in defining the role of HFE in ALS outcomes.

A growing body of evidence suggests that oligogenicity impacts the clinical outcomes in ALS. The co-occurrence of variations in ALS causative and susceptibility genes has been shown to be associated with earlier age at onset^20,21^ and lower survival probability.^73^ In a very recent study, Iacoangeli *et al*. (2025)^74^ have shown that oligogenic carriers had significantly higher disease risk compared to monogenic carriers. However, they did not observe significant relationship was found between oligogenicity and clinical outcomes like age at onset and survival. In our patient cohort, we identified 15.7% of patients with >1 genetic variations. Furthermore, these patients exhibited a significantly delayed age at onset but a shorter survival duration. The presence of additional variants, whether pathogenic, likely pathogenic, or VUS, highlights the potential role of disease-modifier genes in influencing disease pathogenesis. Interaction among these variants may significantly impact disease progression, severity and survival outcomes, contributing to the observed phenotypic heterogeneity.

In conclusion, this study provides valuable insights into the genetic complexity of ALS in the North Indian population, emphasizing the role of both well-established and novel genetic variations in defining clinical outcomes. The study further warrants the investigation into the role of gene–gene interactions and their functional implications in ALS pathogenesis.

### Limitations

Despite its strengths, this study has certain limitations. Firstly, the sample size was relatively small. Secondly, ALS Functional Rating Scale-Revised (ALSFRS-R) scores were not assessed for all patients, which limits the ability to correlate genetic findings with disease severity and prognosis. Additionally, many patients in this part of the country due to socio-economic conditions and remote locations do not come for follow-up, patients diagnosed as ‘Lab Probable ALS’ were excluded.

While WES is a powerful tool to detect SNVs and indels, it does not capture deep intronic variants, CNVs or repeat expansions associated with the disease. Although the repeat expansions of C9ORF72 and ATXN2 were not analysed in the present study, we have previously reported the absence of C9ORF72 expansion in sALS patients from North India (Vats *et al*., 2016). Moreover, while in silico prediction tools aid in variant classification, functional studies are necessary to definitively confirm the pathogenicity of variants, especially for those categorized as VUS or implicated in oligogenic interactions. Addressing these limitations in future studies will enhance our understanding of ALS genetics and its clinical relevance.

## Supplementary Material

fcag003_Supplementary_Data

## Data Availability

The data that support the findings of this study has been included as Supplementary Tables.
