# Supplementary material for: Clinical and genetic determinants of survival in amyotrophic lateral sclerosis patients from North India
Source: Brain Commun. 2026 Jan 8;8(1):fcag003. doi: 10.1093/braincomms/fcag003 (PMC12822497; doi:10.1093/braincomms/fcag003)
Supplement: fcag003_Supplementary_Data [file fcag003_supplementary_data.zip › Supplementary_Figure_1.pdf]

Title: Clinical and genetic determinants of survival in Amyotrophic Lateral Sclerosis patients from North India.

Shiffali Khurana[1,2], Mandaville Gourie-Devi[3,4**], Yuvraj Vats[1], Sagar Verma[1,†], Nirmal Kumar Ganguly[1], Parul Chugh[1], Ankkita Sharma[3], Laxmi Khanna[3], Uma Dhawan[2**], Vibha Taneja[1*]

[1]*Department of Biotechnology and Research, Sir Ganga Ram Hospital, Delhi, India*
[2]*Department of Biomedical Science, Bhaskaracharya College of Applied Sciences, University of Delhi, Delhi, India*
[3]*Department of Neurophysiology, Sir Ganga Ram Hospital, Delhi, India*
[4]*Department of Neurology, Sir Ganga Ram Hospital, Delhi, India*
†*Present Address:*
Department of Biochemistry and Biophysics, Stockholm University, Stockholm, Sweden
Department of Cell and Molecular Biology, Karolinska Institutet, Stockholm, Sweden

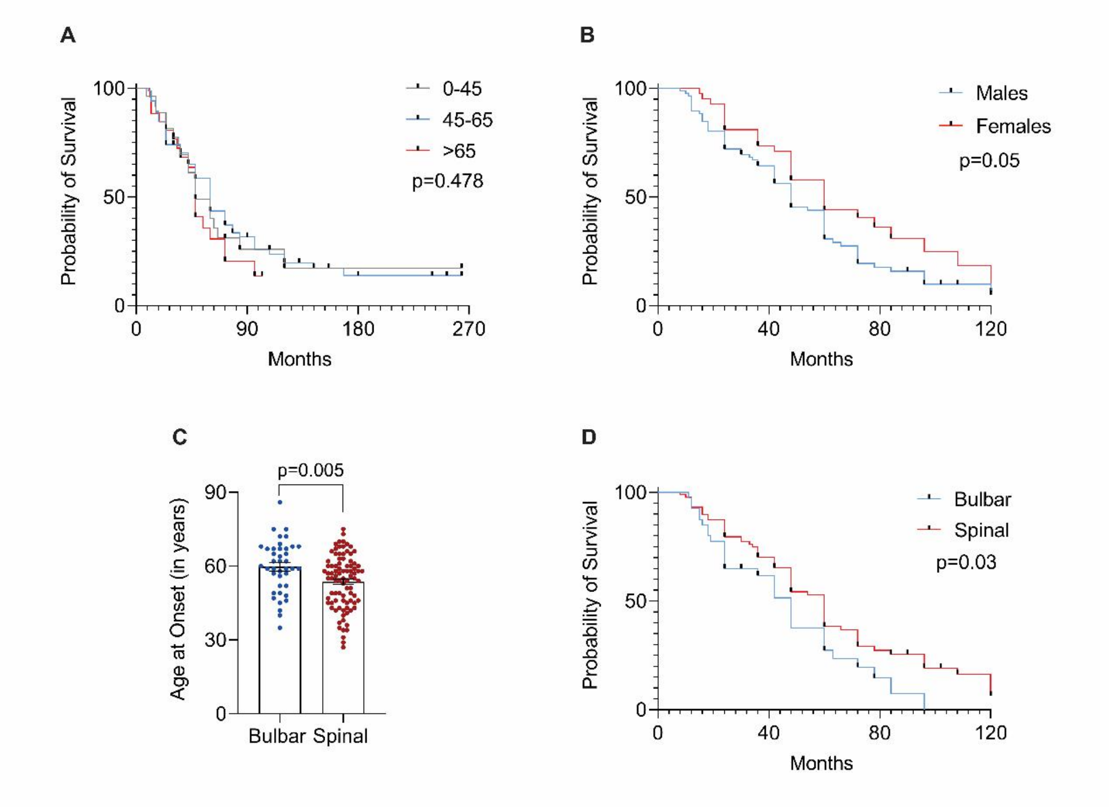

Supplemental Figure 1 Correlation of clinical parameters with demographic features (**A**) Kaplan-Meier survival curves for patients categorized by age at onset, i.e., 0-45, 46-65 and >65 years (**B**) Kaplan-Meier survival curves for patients based on sex (**C**) Correlation of patients based on age at disease onset and site of onset (D) Kaplan-Meier survival curves for bulbar-onset vs spinal-onset patients.

Description of tests used for statistical analysis. (**A**) age 0-45 years n = 27, age 46-65 years n = 85, age >65 years, n = 26, *P* = 0.478 in log-rank test (**B**) Males n = 86, Females n = 42, *P* = 0.05 in log-rank test (**C, D**) Bulbar n = 40, Spinal n = 88, *P* = 0.005 in Mann-Whitney U-test and *P* = 0.03 in log-rank test. Mann-Whitney U-test was used for two group comparisons. Kaplan–Meier curves and log-rank tests were applied to determine the effect of demographic or clinical parameters on survival. A *P*-value of ≤ 0.05 was considered as statistically significant.
